# Prognostic Value of Peripheral Whole Blood Cell Counts Derived Indexes in Gallbladder Carcinoma: A Systematic Review and Meta-Analysis

**DOI:** 10.3389/fonc.2021.707742

**Published:** 2021-06-28

**Authors:** Bowen Xu, Zhiqiang Chen, Jing Zhang, Jianhua Chang, Wei Zhao, Zhaoru Dong, Xuting Zhi, Tao Li

**Affiliations:** ^1^ Department of Hepatobiliary Surgery, General Surgery, Qilu Hospital, Cheeloo College of Medicine, Shandong University, Jinan, China; ^2^ Department of Surgery, Yidu Central Hospital of Weifang City, Weifang, China; ^3^ Department of Hepatobiliary Surgery, The Second Hospital of Shandong University, Jinan, China

**Keywords:** gallbladder carcinoma (GBC), neutrophil-to-lymphocyte ratio (NLR), lymphocyte-to-monocyte ratio (LMR), platelet-to-lymphocyte ratio (PLR), prognosis

## Abstract

**Background:**

Gallbladder carcinoma (GBC) is a rare gastrointestinal malignancy with poor prognosis. Adequate pre-treatment prediction of survival is essential for risk stratification and patient selection for aggressive surgery or adjuvant therapeutic strategy. Whole blood cell count (WBCC) derived indexes are broadly used as prognosticative biomarkers in various cancer types, but their utility in GBC needs to be validated.

**Methods:**

An extensive literature review was conducted in line with PRISMA guideline until June 31 2020, to identify original studies concerning WBCC-derived indexes as prognostic indicators in GBC. All relative parameters were extracted and pooled for statistical analyses.

**Results:**

Fourteen studies incorporating 2,324 patients were included with a high quality and low risk of biases. All 14 studies evaluated the prognostic value of NLR showing a significant correlation with OS in GBC patients (HR = 1.94, *P <*0.001). Elevated NLR was revealed to correlate with TNM stage (stages III and IV, OR = 4.65, *P <*0.001), tumor differentiation (OR = 2.37, *P <*0.042), CA 19-9 (SMD = 0.47, *P* = 0.01), but no significance was found with age, sex and CEA. Positive indicative value of MLR and PLR were also confirmed with a HR of 2.06 (*P <*0.001) and 1.34 (*P <*0.001), respectively.

**Conclusion:**

The WBCC-derived indexes including NLR, MLR/LMR and PLR were validated to be useful prognostic parameters for predicting survival outcomes in GBC patients. These series of indexes, especially NLR, could improve risk stratification and facilitate better patient selection for surgical resection or aggressive chemotherapy in the decision making of GBC patients.

## Introduction

Gallbladder carcinoma (GBC) is a relatively rare gastrointestinal malignancy with an estimated incidence rate about 1.0–3.0/100,000 in the United States and China (1, 2). It is unique in its characteristics that if diagnosed at an early stage (T1a and T1b, before invading beyond gallbladder mucosa), a nearly 100% 5-year survival could be achieved after surgery, but at its later stage (T2 especially T3 and T4, tumor penetrating beyond muscular layer), the long-term survival becomes dismal with only 25% of patients who could undergo potentially curative surgery and just 12–23% could survive for more than 5 years (3, 4). At present, no consensus has been achieved on the optimal treatment of GBC, and multidisciplinary therapy of surgery combined with adjuvant therapy may play a better role in prolonging the survival of patients with advanced GBC (3, 5).

Pathologic TNM staging from the American Joint Committee on Cancer (AJCC) is now the most widely adopted accurate and effective prognosis predicting system for various cancers including GBC (6). However, it could only be assessed after surgery which may account for only a minority of patients suffering GBC. Therefore, it is necessary to pursue preoperative biological markers that could predict survival outcomes of patients, aid in risk stratification and personalized decision making on whether patient could get potential benefits from more aggressive therapies (3).

There is growing evidence that systemic inflammation response (SIR) plays an important role in cancer development and progression, thus various SIR-related biomarkers have been developed and evaluated as prognostic indicators in different cancer types (7, 8). One series of SIR-related biomarkers is derived from peripheral whole blood cell counts (WBCC) which include neutrophil-to-lymphocyte ratio (NLR), lymphocyte-to-monocyte ratio (LMR) or monocyte-to-lymphocyte ratio (MLR), and platelet-to-lymphocyte ratio (PLR). These WBCC-derived indexes showed some great advantages over other pathology related markers, such as easy to perform, good replicability, low cost and preoperative application etc. (7, 8).

Despite the robust and growing data regarding the utility of these WBCC-derived indexes, findings are not uniform across all publications. Furthermore, previous studies mainly focused on colorectal, pancreatic, prostate, lung, esophageal-gastric and breast cancers (7), only few studies conducted investigations on GBC until recently (9–22). Based on the available data, we aimed to systematically review and rationalize the evidence for the prognostic value of these WBCC-derived indexes in predicting outcomes of GBC patients.

## Materials and Methods

This study was conducted in line with the PRISMA and AMSTAR guidelines that were well defined protocols for systemic reviews and meta-analysis (23, 24).

### Inclusion and Exclusion Criteria

The inclusion criteria of studies in this meta‐analysis were defined as: (i) randomized controlled trials (RCT), cohort studies or case control studies; (ii) patients diagnoses of GBC were confirmed by pathology; and (iii) any of the WBCC-derived markers (including NLR, PLR, MLR or LMR) were investigated objects of the studies, and clear cut-off values were given or could be extracted from the Kaplan–Meier curves. The exclusion criteria were: (i) basic researches or animal trials; (ii) abstracts, meta-analysis, case reports, letters, expert comments or reviews; (iii) hazard ratio (HR) investigating correlation between prognostic markers and overall survivals unavailable or cannot be extracted from the K–M curve; and (iv) patients suffered from other primary tumors or with severe infections.

### Search Strategy

PubMed, Medline, Web of Science, Scopus, CNKI, and China Biology Medicine disc (CBMdisc) were searched by two independent researchers from inception to June 31 2020 without any other limits. The medical subject headings (MeSH) terms and free text terms were used to locate articles, combined with the Boolean operators to make an appropriate search strategy. The MeSH terms included “Gallbladder Neoplasms”, “Lymphocytes”, “Neutrophils”, and the free text terms included “Neutrophil-lymphocyte (Ratio)”, “Neutrophil (to) Lymphocyte (Ratio)”.

### Quality Assessment and Data Extraction

After eliminating duplicates, two researchers read titles and abstracts, then by reading full-texts to identify eligible literatures that met the inclusion and exclusion criteria for meta-analysis. The qualitative assessment of RCTs was based on Cochrane risk of bias tool. The Newcastle–Ottawa Scale (NOS) was used to assess risk of bias in non-RCTs. Two researchers used standardized Excel sheets to extract information from the final included studies, including basic information of the study (title, first author, year of publication, study types and number of patients), demographics (patient age and gender), oncology information (tumor types, predominant treatment arms, follow-up time, disease-free survival (DFS), overall survival (OS), hazard ratio (HR) and 95% confidence interval (95% CI), NLR, PLR, LMR and other tumor markers). In the absences of vital data from a study, the corresponding author of the study was inquired by email.

### Statistical Analysis

In this meta-analysis, we mainly focused on the efficacy of NLR, MLR/LMR and PLR on predicting patient survival, and HR with 95% CI was employed to make analysis, as HR incorporates the impact of time-to-event outcomes and is more reliable to reflect survival status of patients over other statistical measures (25). Engauge Digitizer (version 10.8) and method described by Tierney et al. was used to calculate HR from available statistics and Kaplan–Meier curves if the included studies did not provide HR (26). The odds ratio (OR) was chosen to evaluate the association between NLR, MLR/LMR, PLR and clinical features. The numerical data were expressed as means ± standard deviations (SD), and was calculated by using the method from Wan et al. and Luo et al., if the original studies only provided medians and interquartile ranges (27, 28). Heterogeneity between studies was evaluated by Chi-squared (χ2) Q test, and the extent of heterogeneity was quantified by I^2^ index. The random-effects model was applied when the heterogeneity was low (I2 <50%), otherwise, the fixed effects model was adopted (I2 >50%). In addition, sensitivity and subgroup analyses were used to find the source of heterogeneity. The publication bias was assessed by Begg’s funnel plot and Egger’s test. All the statistical data were analyzed with STATA 12.0 (Version 12.0, Stata Corp LP, College Station, TX). A value of *P <*0.05 was considered statistically significant.

## Results

### Search Results and Study Characteristics

**Figure 1:**
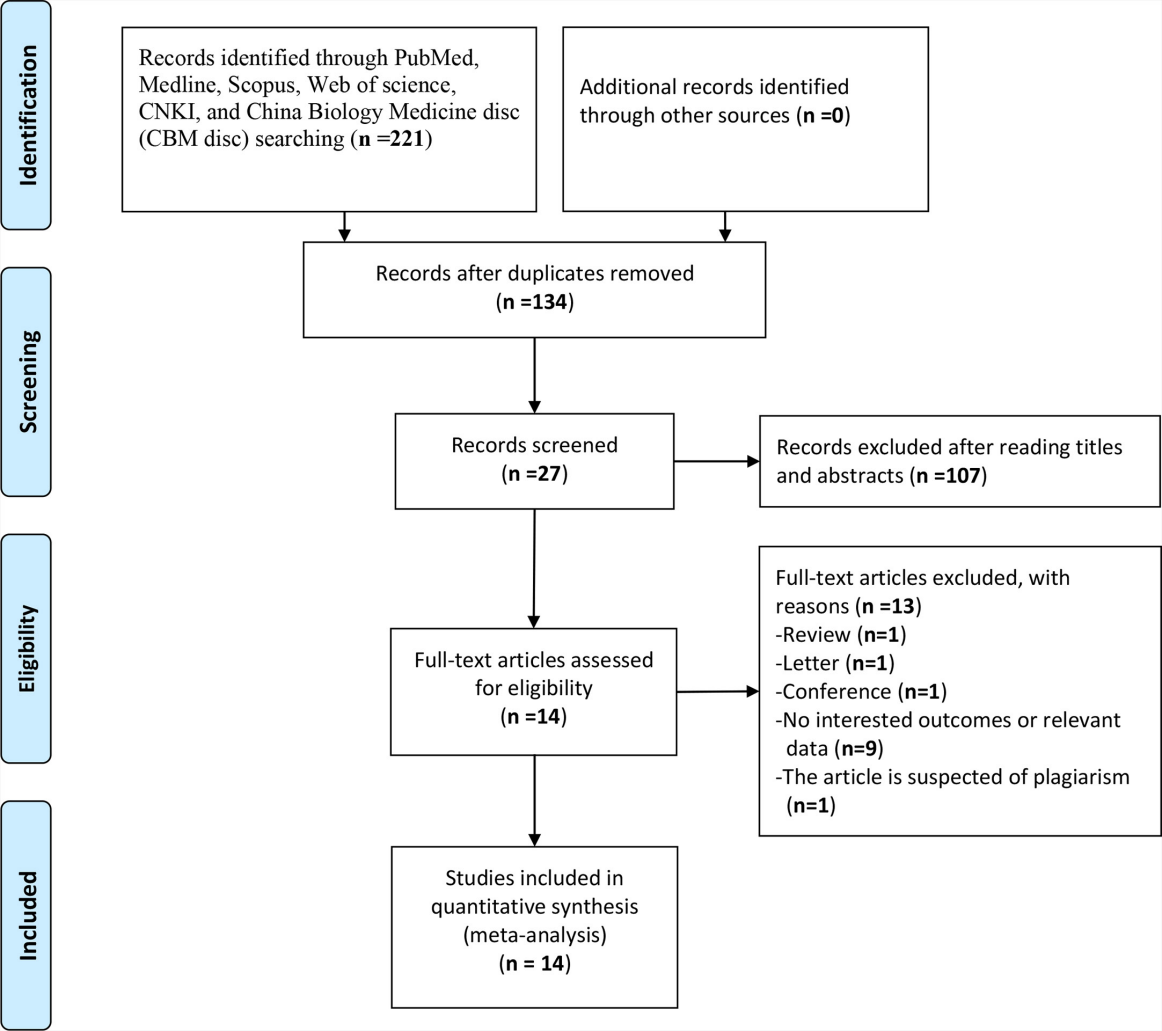
Flow diagram following the PRISMA template of the search strategy for studies included in this meta-analysis.

The study selection was carried out in accordance with PRISMA flowchart (**Figure 1**). A total of 221 potentially relevant studies were identified from six databases by using the formulated search strategy. After removal of duplicates, we browsed the titles and abstracts of the remaining 134 studies, and 107 studies were excluded according to the inclusion and exclusion criteria. Then we assessed full-text review of the remaining 27 studies, another 13 studies were excluded and finally 14 retrospective cohort studies were included for meta-analysis (9–22). Basic characteristics of included studies were listed in **Table 1**. There were 2,324 patients in total from all studies with a mean NOS score of 7.6 ± 0.69, indicating a low risk of bias. NLR was evaluated by all the 14 studies enrolled, while MLR/LRM and PLR were investigated by only six and eight studies, respectively. Although we could calculate or directly withdraw HR data from all studies, it needs to be noted that different cut-off values were employed, as well as calculation methods for cut-off value among which ROC curve analysis was the mostly adopted by majority of authors.

**Table 1:** Basic characteristics of included studies.

Study	Country	Patients (n)	Sex (M/F)	TNM stage (n)	cut-off value with HR (*P* value)			calculation method for cut-off value	NOS
					NLR	MLR/LMR	PLR		
Wu (9)	China	85	NR	I/II/III/IV (21/13/47/6)	2.3 1.77 (0.016)	NR	NR	median value of effect size	9
Gao (10)	China	90	47/43	I/II/III/IV (54/11/23/2)	5 3.09 (0.027)	NR	NR	refer to others	8
Zhang (11)	China	145	68/77	I/II/III/IV (7/12/75/51)	1.94	NR	113.3	ROC curve	8
					2.73 (0.001)		1.74 (0.001)		
Beal (12)	America	187	NR	NR	5	NR	NR	refer to others	8
					3.52 (0.02)				
Zhang (13)	China	316	215/101	I–II/III–IV (28/288)	2.61	NR	NR	ROC curve	8
					1.65 (0.008)				
Cui (14)	China	159	NR	I/II/III/IV (13/27/50/69)	4.39	0.30/NR	181	ROC curve	8
					1.57 (0.01)	1.61 (0.006)	1.24 (0.23)		
Tao (15)	China	84	28/56	III/IV (35/49)	3.2	0.25/NR	117.7	ROC curve	9
					2.348 (0.002)	2.42 (0.001)	1.859 (0.024)		
Du (16)	China	220	122/98	NR	5.1	NR/2.92	178	X‐tile software	7
					1.38 (0.62)	0.69 (0.03)	0.75 (0.44)		
Choi (17)	South Korea	178	95/83	III/IV (39/139)	2	0.24/NR	108	refer to others	9
					2.06 (0.001)	2.53 (0.001)	1.69 (0.019)		
Deng (18)	China	169	55/114	I/II/III/IV (16/37/76/40)	2.61	NR/2.66	145.3	ROC curve	8
					3.30 (0.008)	1.55 (0.027)	1.221 (0.376)		
Liu (19)	China	90	NR	I–II/III–IV (20/70)	4.33	NR	NR	mean value of effect size	7
					3.84 (0.01)				
Navarro (20)	South Korea	197	83/114	II/III/IV (148/41/8)	2.4	NR/4	148	ROC curve	8
					1.80 (0.44)	1.25 (0.739)	0.53 (0.432)		
You (21)	South Korea	173	87/86	III/IV/IV (1/8/164)	3	NR	190	refer to others	8
					1.65 (0.017)		1.19 (0.405)		
Mady (22)	America	231	72/159	NR	5	NR	NR	refer to others	9
					1.70 (0.003)				

TNM, tumor/node/metastasis stages; HR, Hazard Ratio; NLR, neutrophil-to-lymphocyte ratio; MLR, monocyte-to-lymphocyte ratio; LMR, lymphocyte-to-monocyte ratio; PLR, platelet-to-lymphocyte ratio; NR, not reported; ROC, receiver operating characteristic; NOS, Newcastle–Ottawa Scale.

### Prognostic Value of NLR and Its Association With Clinical Features

**Figure 2:**
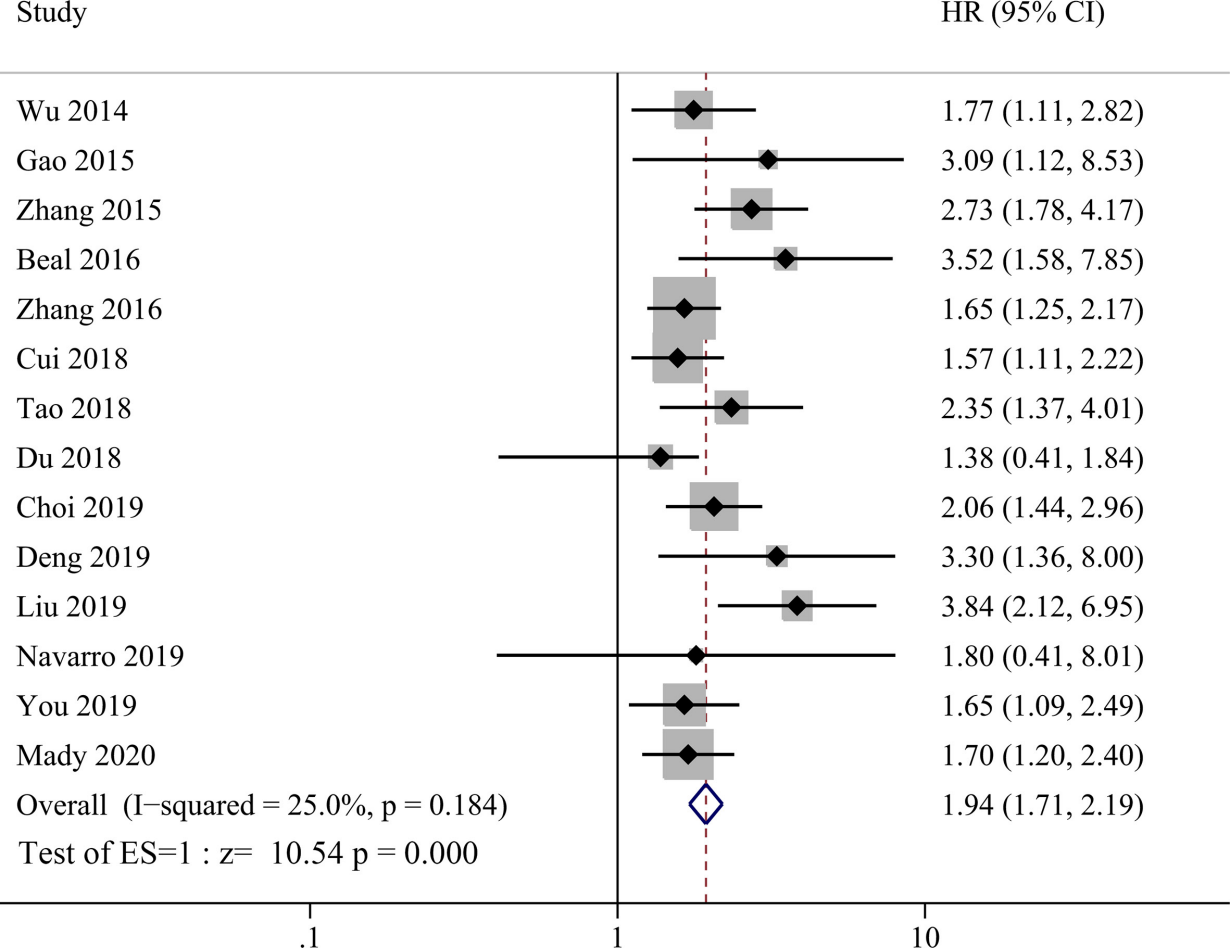
Forest plot for the association between neutrophil-lymphocyte ratio (NLR) and overall survival of patients with Gallbladder carcinoma (GBC).

NLR was evaluated in all 14 studies enrolled and HR data could be extracted directly from 14 studies and calculated from K–M curve from one study using the method as described above (26). The heterogeneity was not significant (*P* = 0.184, I^2^ = 25%) among studies, so the fixed effect model was used for meta-analysis evaluating prognostic value between high NLR and low NLR groups. Compared with lower NRL group, higher pretreatment NLR was significantly correlated with shorter OS in GBC patients as shown in **Figure 2** with a HR value of 1.94 (95% CI=1.71–2.19, *P <*0.001).

As most included studies investigated the association between NLR and various clinical parameters including TNM stage, tumor differentiation, CA199 and CEA etc., we then summarized the pooled OR of these parameters. As shown in **Table 2**, elevated NLR had significant correlation with TNM stage (stages III and IV, OR = 4.65, 95% CI = 1.96–11.03, *P <*0.001), tumor differentiation (OR = 2.73, 95% CI = 1.04–7.18, *P <*0.042), CA 19-9 (SMD = 0.47, 95% CI = 0.11–0.82, *P* = 0.01), but no significance was found with age, sex and CEA. Due to the significant heterogeneity between studies, random-effects models were used for analysis.

**Table 2:** The association between elevated neutrophil-to-lymphocyte ratio (NLR) and clinical features.

Clinical parameter	Number of studies	Number of participants	Pooled results		*P* value
			Effect Size	95% CI	
Age (>60 years)	2	180	OR: 1.17	0.45–3.03	0.29
Age (>65years)	2	243	OR: 0.62	0.25–1.51	0.749
Gender (Male)	9	1,378	OR: 1.33	0.95–1.87	0.099
CEA (High)	3	320	SMD: 0.025	−0.198–0.249	0.826
CA-199 (High)	4	498	SMD: 0.47	0.11–0.82	0.01
TNM stage (III and IV)	7	969	OR: 4.65	1.96–11.03	<0.001
Tumor differentiation (Poor and undifferentiated)	5	726	OR: 2.73	1.04–7.18	0.042

OR, odds ratio; CI, confidence interval; CEA, carcinoembryonic antigen; CA-199, carbohydrate antigen 199; TNM, tumor/node/metastasis stage; SMD, Standard mean difference.

### The Prognostic Value of MLR/LMR

**Figure 3:**
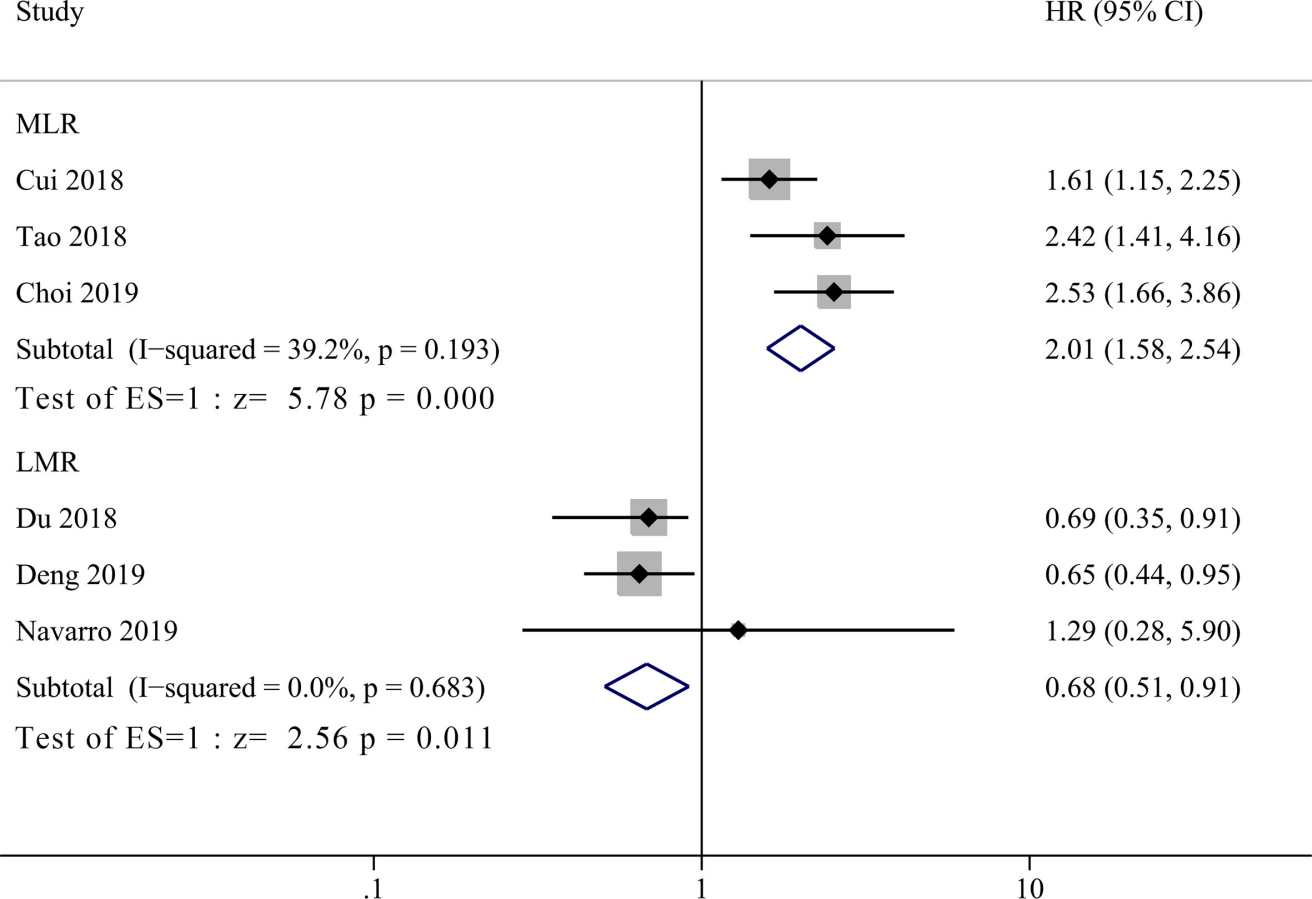
Forest plot for the association between lymphocyte-to-monocyte ratio (LMR) or monocyte-to-lymphocyte ratio (MLR) and overall survival of patients with Gallbladder carcinoma (GBC).

Three studies incorporating 421patients reported the prognostic value of MLR in GBC patients (14–16). As shown in **Figure 3**, high MLR was significantly correlated with shorter OS in GBC patients compared to low MLR group, with a pooled HR of 2.06 (95% CI = 1.51–2.82, *P <*0.001). The heterogeneity was not significant (*P* = 0.193, I^2^ = 39.2%) among studies, and the fixed effect model was used for meta-analysis. Another three studies enrolling 586 patients reported the relationship between LMR and OS in GBC patients (17, 18, 21). The primary results showed that there was no significant correlation between LMR and prognosis of GBC patients with a HR of 1.08 (95% CI = 0.58–2.07, *P* = 0.814, I^2^ = 70%). Due to the high heterogeneity, we performed sensitivity analysis and revealed that the heterogeneity decreased significantly after excluding the study by Deng et al. (17), but the final result did not change either (HR = 0.73, 95% CI = 0.46–1.15, I^2^ = 0%, *P* = 0.176). By re-reading the study by Deng et al., we found that they set the lower LMR group as experimental group instead of control group as the other two enrolled studies (18, 21). Therefore, we re-calculated the reciprocal of HR and 95% CI with a correction of pooled HR of 0.68 (95% CI = 0.51–0.91, I^2^ = 0%, *P* = 0.011), showing significant correlation between low LMR and poor OS in GBC patients.

### The Prognostic Value of PLR

**Figure 4:**
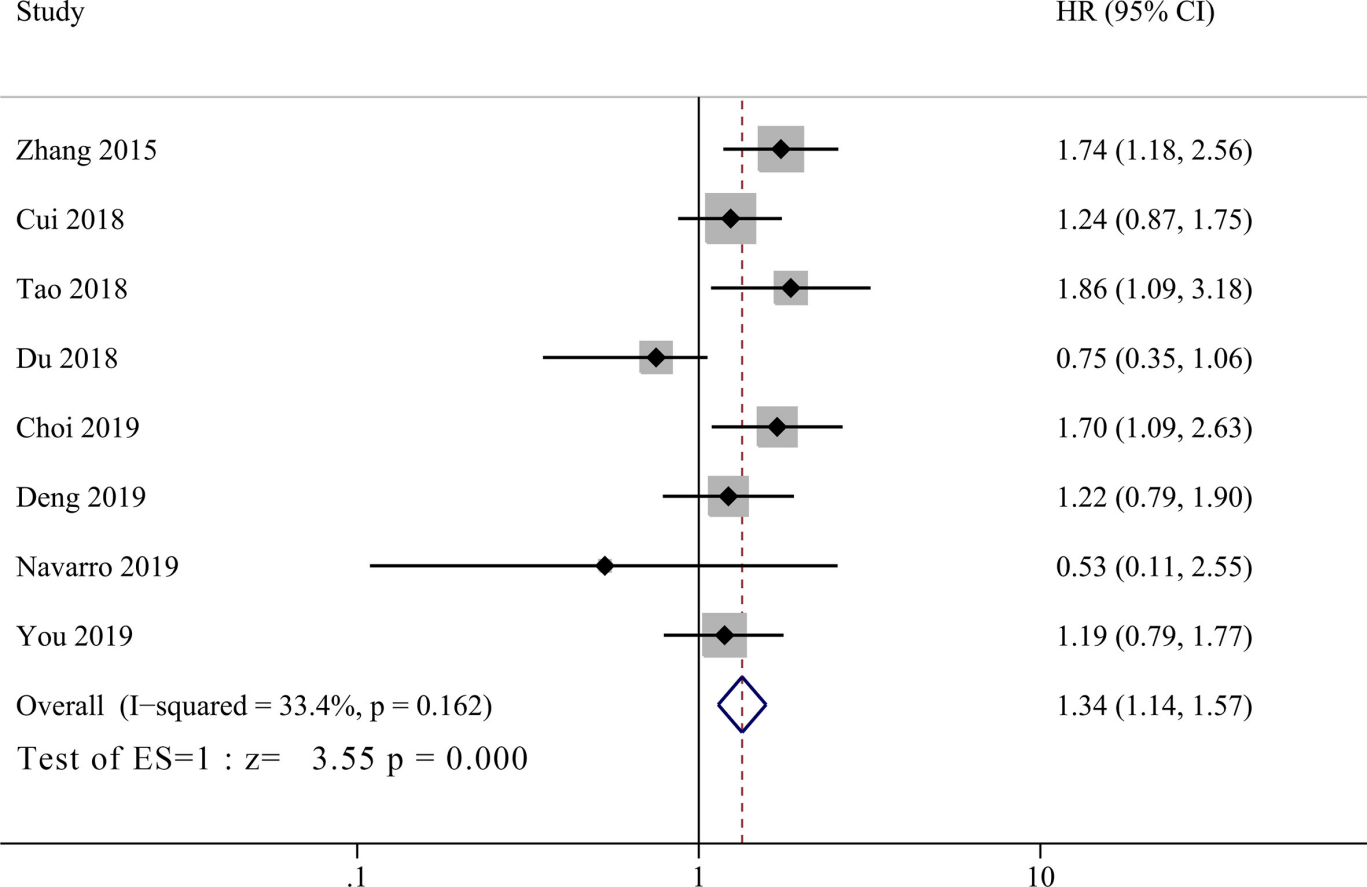
Forest plot for the association between platelet-to-lymphocyte ratio (PLR) and overall survival of patients with Gallbladder carcinoma (GBC).

Eight studies incorporating 1,325 patients investigated prognostic value of PLR in GBC patients (11, 14–18, 20, 21). No significant heterogeneity between groups was observed (I^2^ = 33.4%, *P* = 0.162), and the pooled HR showed that low PLR group had significant better OS than high PLR group (**Figure 4**, HR = 1.34, 95% CI = 1.14–1.57, *P <*0.001).

### Subgroup Analyses and Publication Bias

**Figure 5:**
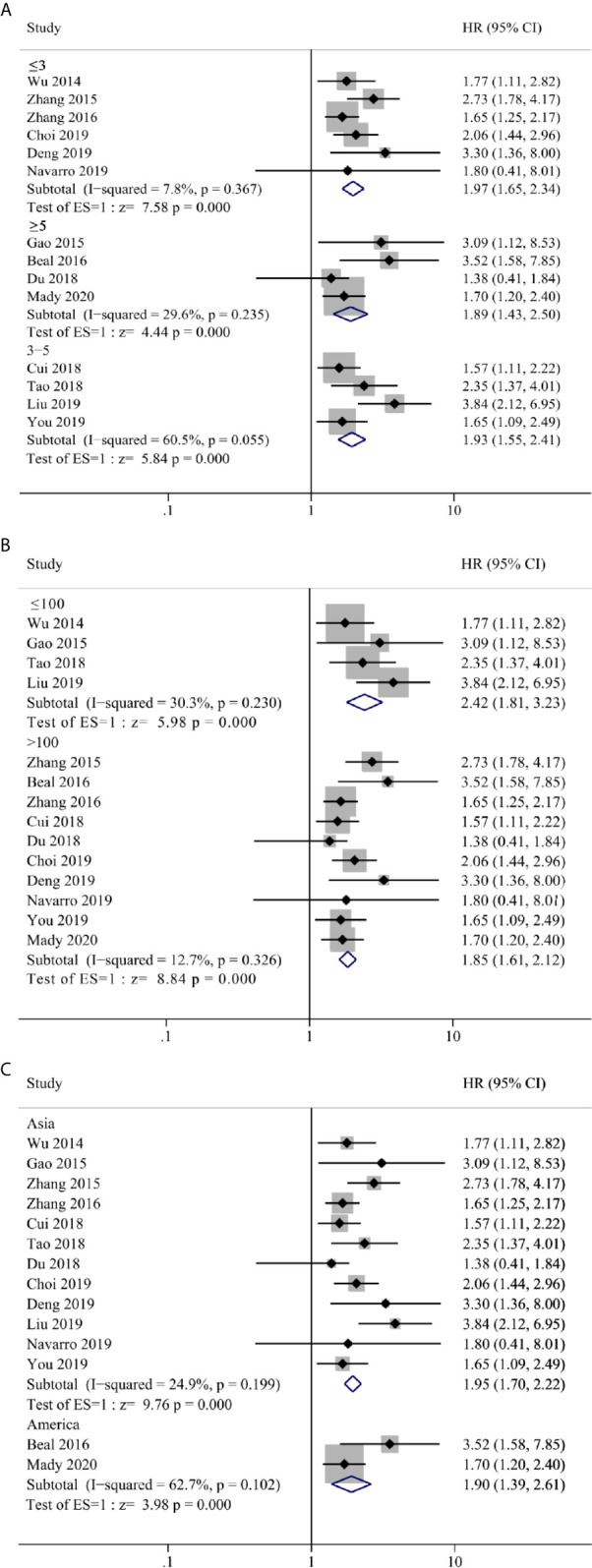
Forest plot and subgroup analysis for **(A)** cut-off value, **(B)** sample size and **(C)** geographic area of the correlation between neutrophil–lymphocyte ratio (NLR) and overall survival of patients with Gallbladder carcinoma (GBC).

Our meta-analyses result above confirmed that NLR, LMR/MLR and PLR could be used as prognostic predictor of OS in GBC patients. Although there was no significant heterogeneity between groups, we still performed subgroup analysis in case of patient selection bias. Three stratification parameters were selected for subgroup analysis, that include cut-off value (≤3, 3–5 and ≥5), sample size (>100 and ≤100) and geographic area (Asian and America). Due to sample size and data availability, the subgroup analysis was only performed in NLR group, which confirmed a positive result as ungrouped analysis (**Figure 5**).

**Figure 6:**
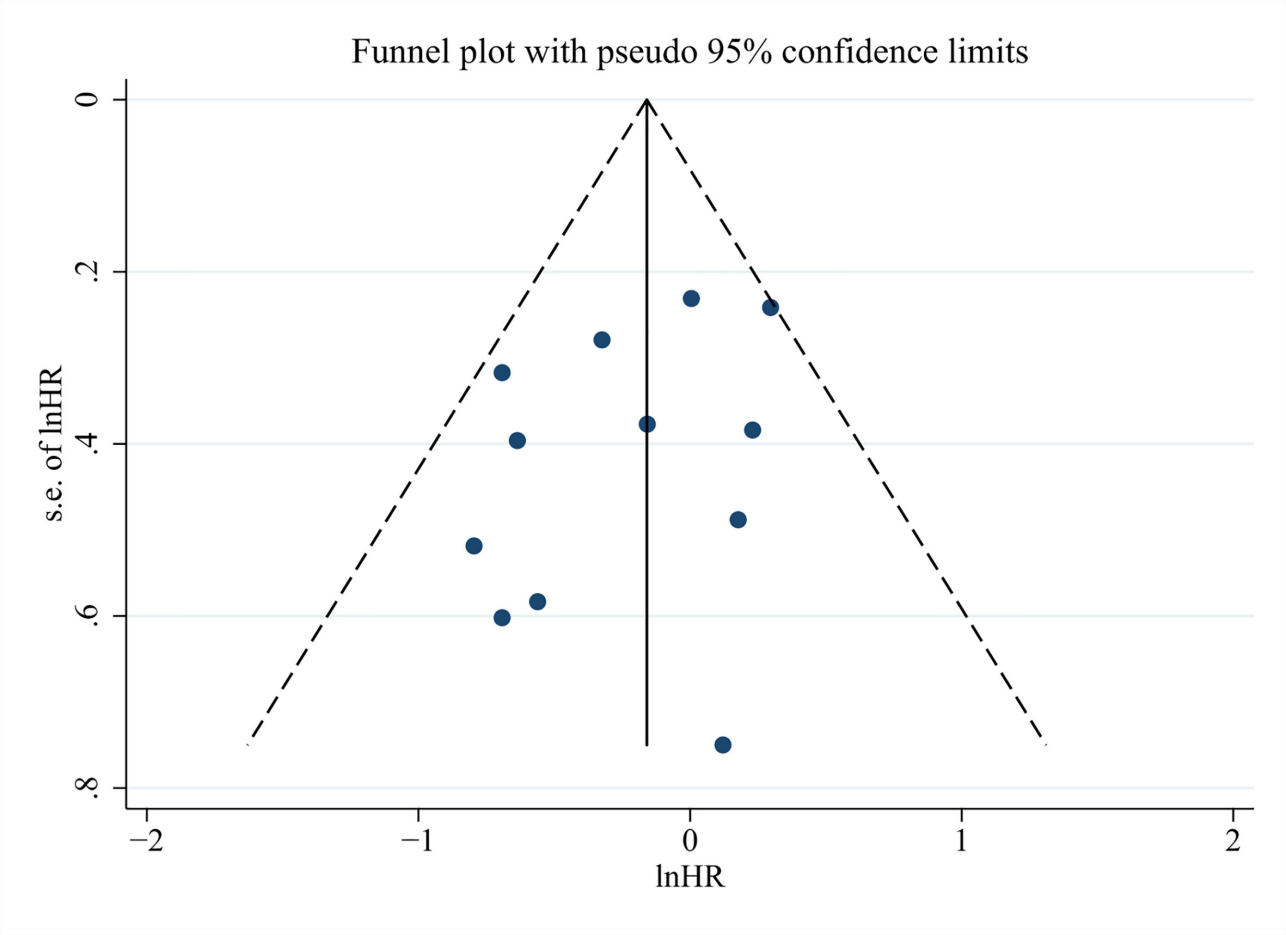
Funnel plots for detecting publication bias of the association between neutrophil–lymphocyte ratio (NLR) and overall survival of Gallbladder carcinoma (GBC).

In the end, we run Begg’s and Egger’s test to examine the main effect indicators of this study. The results showed that there was no significant publication bias among included studies, and the funnel plot was symmetrical (**Figure 6**, Begg’s test *P* = 0.373, Egger’s test *P* = 0.225).

## Discussion

GBC is rare and one of the most aggressive cancers with poor prognosis worldwide (29). Up to date, pathological TNM (pTNM) staging is still the gold standard risk stratification system and reversely correlated with survival of GBC patients, but with a limitation of being only assessable after surgery (6). Surgery still remains the only potentially curative therapy, but only a minority of patients has the chance of getting radical operation and adjuvant therapy still has its position in GBC treatment (3, 30). Thus, efficient pre-operative or pre-treatment parameters/indexes for prediction of prognosis should be pursued, as they may help identifying patients who might benefit from more aggressive adjuvant therapies (31, 32).

There are some kinds of predictive parameters/indexes advocated by different authors to be used for pre-treatment evaluation of survival in various cancer types (21, 33–39). These markers were derived mainly on the basis of three major groups of clinical parameters, specifically reflecting nutrition status (glucose, albumin and cholesterol), immune status (lymphocyte, monocyte), inflammation status (neutrophil, platelet and C-reactive protein) and their cross combinations. Examples may include GLR (glucose to lymphocyte ratio) (21), CONUT (controlling nutritional status score, calculated from albumin, lymphocyte and cholesterol) (34), PNI (prognostic nutritional index, calculated from albumin and lymphocyte) (35), GPS (Glasgow prognostic score, calculated from C-reactive protein and albumin) (38), SII (systemic immune-inflammation index, derived from platelet, neutrophil and lymphocyte) (39) and so on. Compared to pTNM or other pathology-based evaluators, these markers share similar advantages, such as easy to assess and replicable, low cost and preoperative applicability etc. (7, 8). Although there were growing evidence of using these markers to predict survival in various cancers, no worldwide consensus has been achieved and concerns rise about the efficiency and accuracy of these makers, and thus their clinical utilities are still suspended and limited.

In the present review, we focused on the prognostic value of one series of the most easily accessible and investigated markers that is WBCC-derived indexes including NLR, LMR/MLR and PLR in GBC patients. Our meta-analysis showed that all these indexes could be used as prognostic factors for GBC patients, which was in compliance with results in other cancer types (31). And we also pooled available data together and revealed that elevated NLR was significantly correlated with TNM stage, tumor differentiation and CA19-9, which could explain in part the mechanisms of these indexes being used as prognostic markers for GBC patients.

Although WBCC-derived indexes are certified to be useful parameters for predicting prognosis in various cancer types, the underlying mechanisms largely remains to be elucidated. First of all, the theoretical foundation of their usage as prognostic biomarker lies in that different types of peripheral blood cells could be considered to reflect host immune and inflammation status, which play important role in systemic inflammatory response (SIR), carcinogenesis, tumor microenvironment modulation and progression (40–42). Indeed, inflammatory microenvironment has been proposed as one hallmark of cancer (43), infiltrating immune and inflammatory cells are increasingly accepted to be generic constituents of tumors, and they exert conflicting ways for tumor development: tumor-antagonizing effect as for lymphocytes while tumor-promoting effect as for neutrophils and monocytes (37, 39). More specifically, infiltrating lymphocytes are major antitumor components that may induce cancer cell apoptosis *via* interaction of CD4^+^ and CD8^+^ T cells (44, 45). In contrast, low lymphocytes within or around tumor area may be responsible for an insufficient immune surveillance that leads to tumor progression and inferior survival of patient (44). On the other hand, neutrophils and monocytes play a tumor-promoting role in malignancies. In short, neutrophils, as another key component of SIR, are recruited to tumor area, produce various kinds of cytokines and chemokines that are implicated in promoting tumor progression *via* all kinds of pathophysiological process, such as matrix degradation, immunosuppression, angiogenesis etc. (43, 46, 47). Besides, peripheral monocytes are known for their association with the level of tumor-associated macrophages (TAMs) which could also produce cytokines and enzymes with protumoral functions, including tumor cell migration, invasion, metastasis as well as immunosuppression (48, 49). On the other hand, Deng and collogues revealed in their study that SIR biomarkers were significantly correlated with tumor differentiation, TNM stage or anemia, this could partially explain the positive correlation between SIR biomarkers and prognosis (17).

As we summarized above, most studies concluded with a favorable and positive results on prognosis predictive value of these WBCC-related biomarkers, but there are some limitations in these studies. Firstly, the cut-off value was discrepant through studies and different methods were employed to get it defined (**Table 1**). The receiver operating characteristic (ROC) curve analysis was the mostly adopted method and the accuracy of it was determined by sample size and subjective populations (as parameters vary among different tumors, stage, treatment etc.) (50). So future large volume investigations among different populations should be considered to identify the optimal cut-off values for each index. Secondly, the WBCC parameters are continuous variables which may present quite different values, especially before and after treatment. For example, the neutrophils are more susceptible to antibiotics usage, and may differ greatly from before and after surgery. So dynamic observations at different time point or analysis on trend should be more significant and encouraged for future investigations. Thirdly and finally, it is impossible to have one index fit to all situations, and the prognostic value of each index varies between different tumors, and even between different stages and treatment strategies in same tumor. So, the accuracy and reliability of single index may be challenged and an optimized mathematical model should provide some benefits to solve this issue. For example, Deng and collogues proposed a predictive nomogram using all the significant independent predicators to predict the patient survival (17). Each variable could be assigned a weighted number of points in the model, and the sum of points for each patient could be used to predict prognosis.

There were some limitations in this meta-analysis. Firstly, all studies were retrospective with low quality of evidence, further high quality RCT studies should be designed for future investigations. Secondly, the cut-off value was different through studies. Although we performed sub-group analyses, a single defined cut-off value would provide better comparison between studies. Thirdly, although there was no significant heterogeneity among most included studies, the patient inclusion criteria varied through studies, such as operation method, tumor staging, chemotherapy strategy etc. Due to the limited sample size, subgroup analysis was not practicable. Finally, all the included 14 studies investigated NLR as prognosis marker for GBC, while only few studies investigated MLR, PLR, so the statistics for these latter meta-analyses are under-powered, and future studies on these markers should be expected and included for further meta-analysis.

In conclusion, our meta-analysis validated that WBCC-derived indexes including NLR, MLR/LMR and PLR could be used as prognostic parameters for predicting survival outcomes in GBC patients. These series of indexes, especially NLR, could improve risk stratification and facilitate better patient selection for surgical resection or aggressive chemotherapy in the management of GBC.

## Data Availability Statement

The original contributions presented in the study are included in the article/supplementary material. Further inquiries can be directed to the corresponding authors.

## Author Contributions

Study concepts: TL, ZC and BX. Study design: TL, ZC and BX. Data acquisition: BX, JC, ZC and WZ. Quality control of data and algorithms: BX, JC, ZD and XZ. Data analysis and interpretation: BX, ZC and JC. Statistical analysis: ZC, BX, JZ, JC and WZ. Manuscript preparation: BX, ZC, JZ and JC. Manuscript editing: ZC, BX and JZ. Manuscript review: TL, ZC and BX. All authors contributed to the article and approved the submitted version.

## Funding

This study was supported by the Taishan Scholars Program for Young Experts of Shandong Province (tsqn20161064), the National Natural Science Foundation of China (81874178 & 82073200) for TL.

## Conflict of Interest

The authors declare that the research was conducted in the absence of any commercial or financial relationships that could be construed as a potential conflict of interest.
